# A Case Report of Exfoliative Bullous Rash Following AstraZeneca Vaccine

**DOI:** 10.7759/cureus.31559

**Published:** 2022-11-16

**Authors:** Usman Saleem, Elizabeth S Goh

**Affiliations:** 1 General Internal Medicine, Hampshire Hospital Foundation Trust, Winchester, GBR

**Keywords:** covid-19, vaccine related rash, exfoliative bullous rash, side effect post vaccination, astra zeneca covid-19 vaccine

## Abstract

The coronavirus disease 2019 (COVID-19) pandemic has affected globally; but thanks to vaccines, the effect has been countered with the help of a vaccination programme. We report a case of exfoliative bullous rash in a 69-year-old lady admitted to the acute medicine unit with complaints of rash post-AstraZeneca (AZ) vaccine administration. The rash initially started under her breast and had spread to other parts of the body without mucosal involvement. She was noted to have neutrophilia and raised inflammatory markers and subsequently commenced on antibiotics. Histology showed sub-epidermal blistering but negative immunofluorescence which excluded immune-bullous disease. A significant improvement was seen, and the presentation subsided after treatment with antibiotics and emollient therapy.

## Introduction

Severe Acute respiratory syndrome coronavirus-2 (SARS-CoV-2) infection commonly known as coronavirus disease 2019 (COVID-19) was declared a global health emergency and found to spread in more than 101 countries in the world. Vaccination has proved to be effective in reducing COVID-19 infection. The Global Vaccination Programme was started after vaccines were approved by WHO for emergency use. The Oxford/AstraZeneca Vaccine was declared safe and effective at protecting people from the extremely serious risk of COVID-19 including death and hospitalization and severe disease. It showed an efficacy of 72 % against symptomatic SARS-CoV-2 infection as per initial data from trial participants who received two standard doses with intervals varying from 4 to 12 weeks [1]. Several mild side effects including fever, local inflammation, and pain were reported initially [2]. However, as the vaccination was given to a significant number of populations, very few rare adverse effects have been reported with the Oxford/Astra Zeneca vaccine such as thrombosis with thrombocytopenia syndrome (TTS) which involved a risk of coagulation with low platelet count [2]. There were a few numbers of cases reported as vaccine-induced rash and we wanted to report this rare case presentation which got improved after management.

## Case presentation

**Figure 1:**
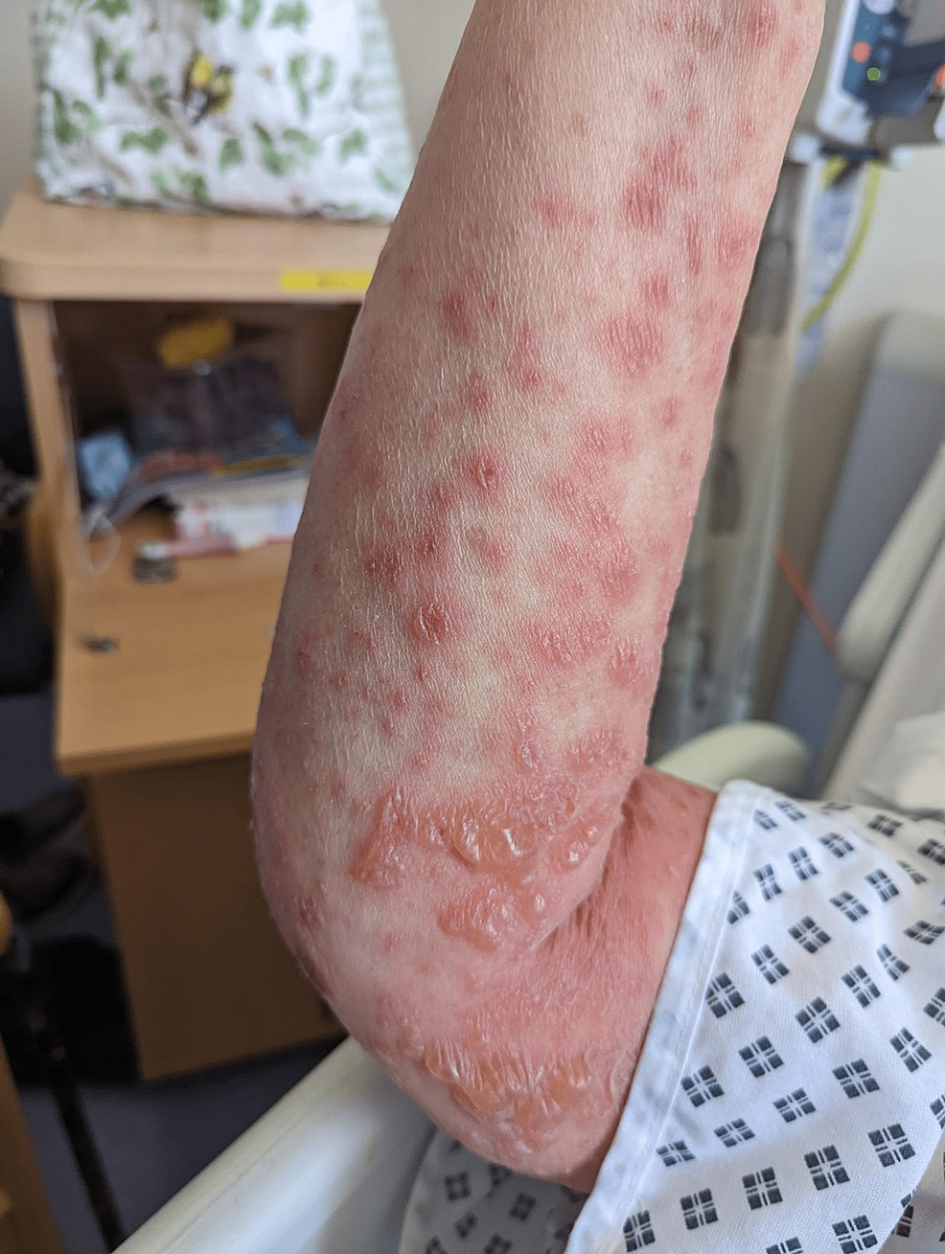
Exfoliative bullous rash presentation

**Figure 2:**
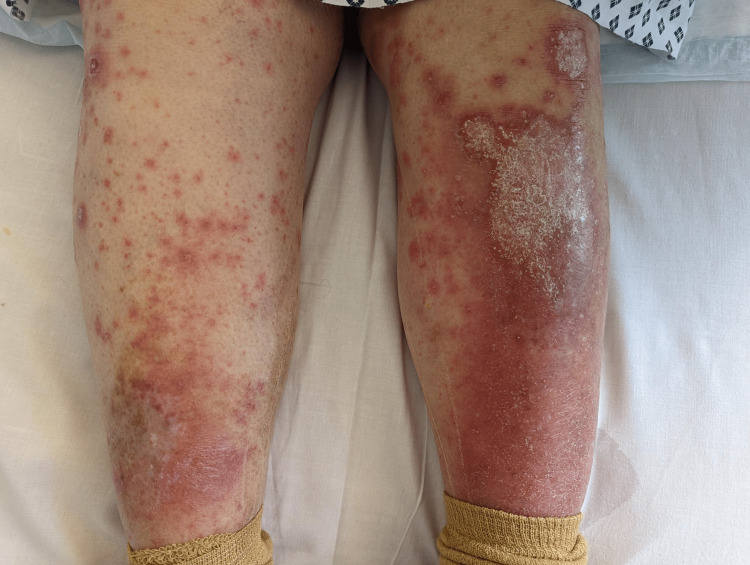
B/L lower limbs involvement with plaques and non-blanching papules B/L: Bilateral

**Figure 3:**
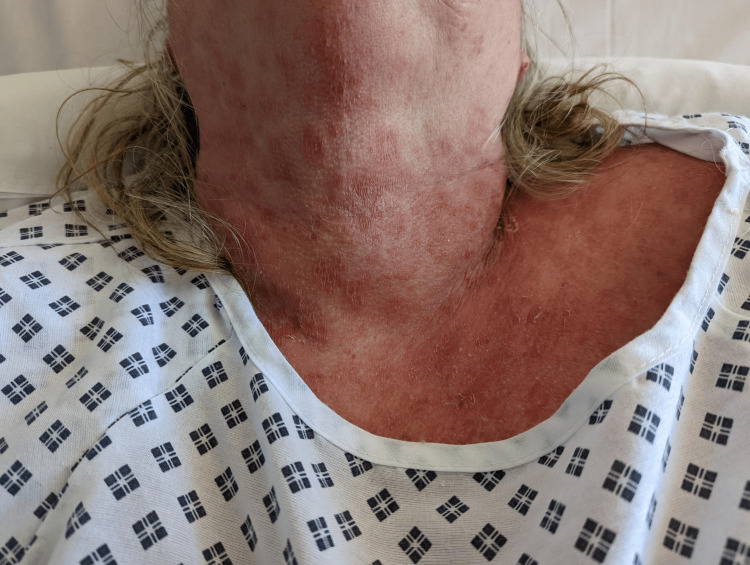
Rash involved neck and face but not oral mucosa

**Figure 4:**
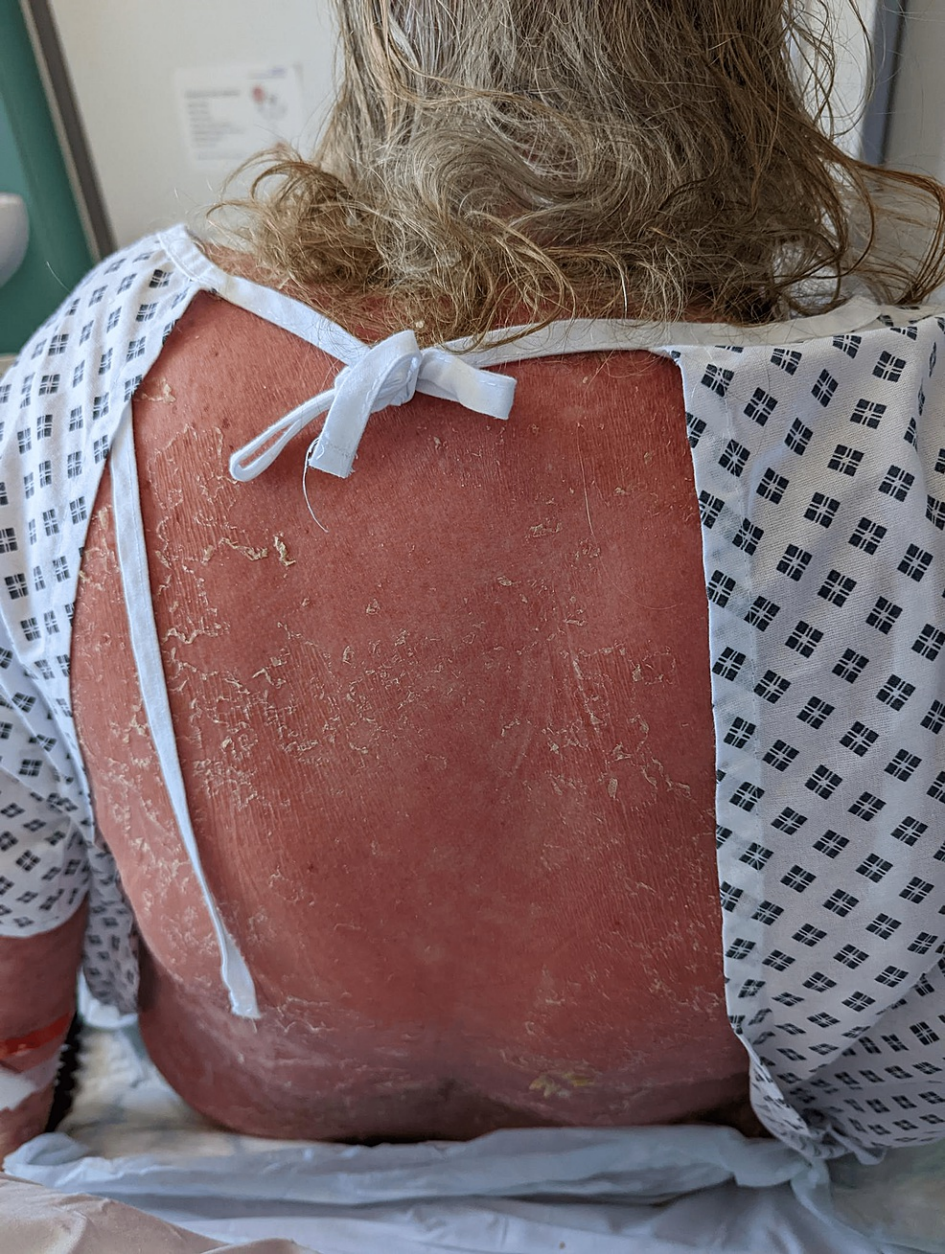
Rash involving back with scalding of the surrounding skin

**Figure 5:**
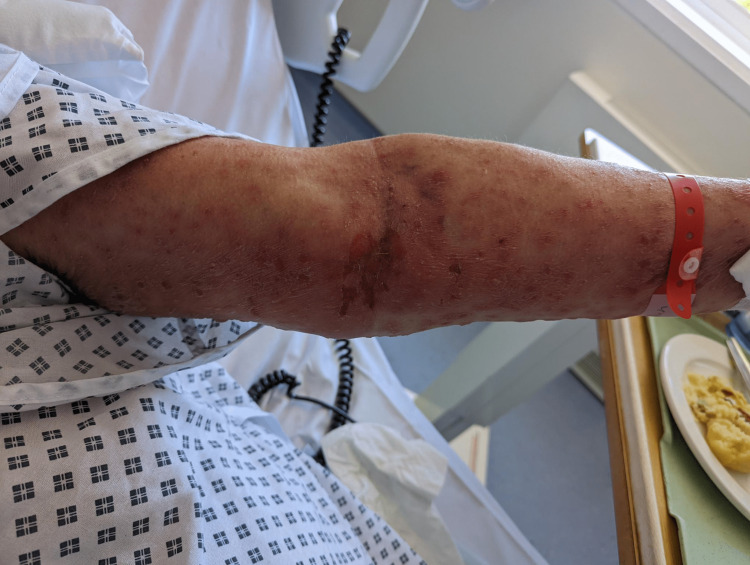
Flexor surface of the arm

**Figure 6:**
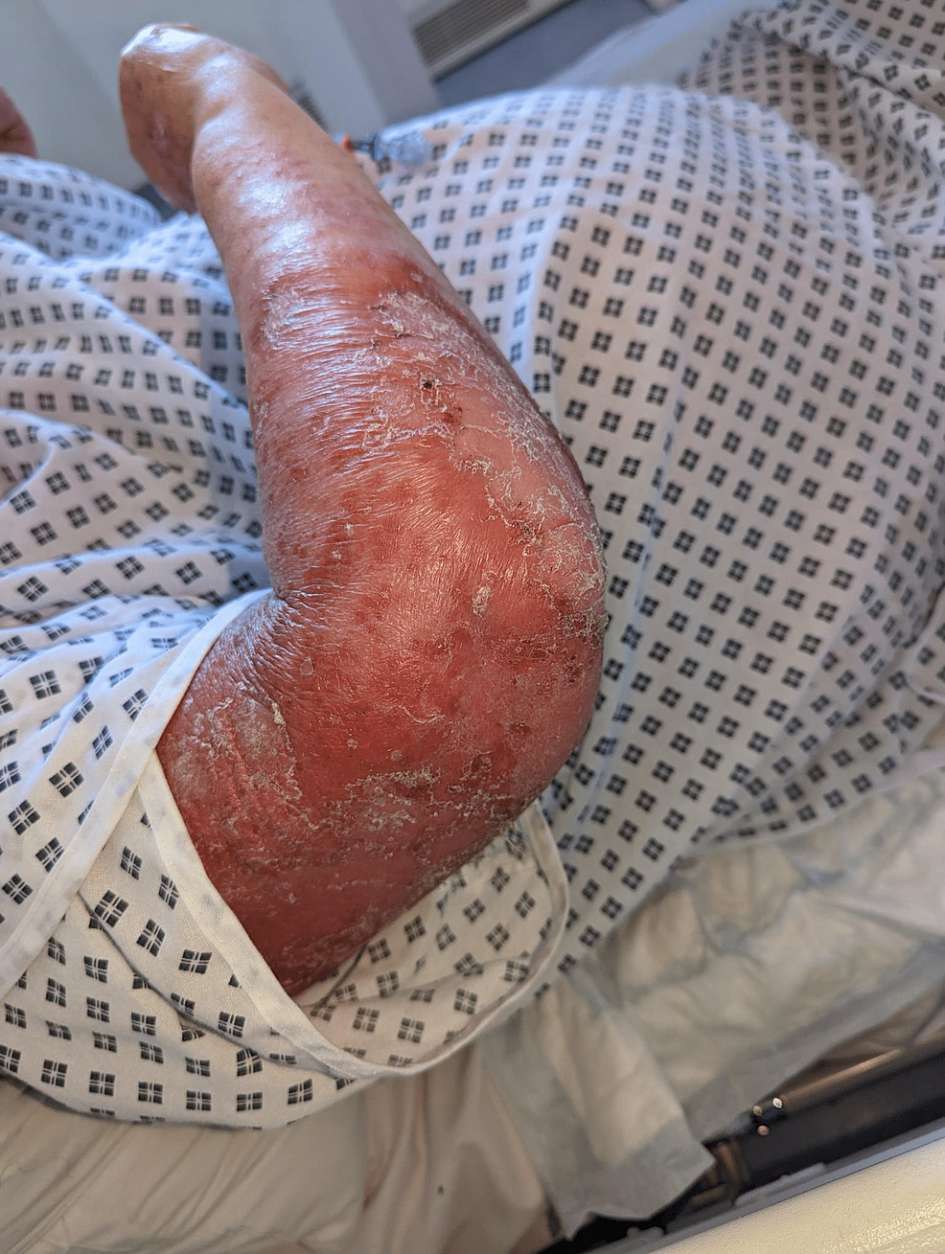
Extensor surface of the arm

A 69-year-old, diabetic female with one month of an undiagnosed, generalized, pruritic, scaly, painless, erythematous rash, unresponsive to topical treatment, developed a macular rash in her inframammary region, within 36 hours after receiving her AstraZeneca booster vaccine. This lesion progressed to her thighs and developed into painful, non-pruritic, plaques, non-blanching papules, and vesicles-some of which had de-roofed, as seen in figures below (Figures 1-6). There was no facial or mucosal involvement.

The patient showed no response to topical antifungals or clindamycin prescribed by her General Practitioner and similarly had no respite whilst on clarithromycin and antihistamines prescribed to her at the Emergency Department. The patient later returned to be admitted with worsening inflammatory markers, liver function tests and hyponatremia along with a new Acute Kidney Injury.

Adding up her poor response to antimicrobials and her clinical presentation, a clinical suspicion of drug-induced vesicular eruption secondary to the AstraZeneca vaccine was entertained. A skin biopsy was arranged, and the histopathology report demonstrated sub-epidermal blistering with negative immunofluorescence not keeping with an immuno-bullous disease. Biopsy reported as with direct immunofluorescence showed accentuation of the junction with immunoglobulin(Ig)G and IgA, but no bright or above-background staining. IgM and C3 were negative. Punch biopsy of skin demonstrated extensive neutrophils in the surface epithelium, below the keratin layer. There was subepidermal oedema and splitting and subepidermal vesicle formation which contains scattered chronic inflammatory cells. Within the dermis, there was a mixed perivascular inflammatory infiltrate. Eosinophils were inconspicuous. Fungal stain was negative. The frozen section H&E showed superficial pustules, acute inflammation in the epidermis and oedema at the dermo-epidermal junction with incipient splitting.

Her clinical improvement eventually became evident after being treated with oral doxycycline and regular application of shower emollients, double base and aggressive IV fluids. There was a marked improvement noted in her inflammatory markers (CRP improved from 350 to 39).

The patient showed great response to management and skin exfoliation was improved. In view of her improvement, she was discharged from the medical ward with further follow-up with dermatology in the clinic.

## Discussion

The AstraZeneca (AZ) vaccine also known as Vaxzevria and Covidshield was approved for use on 30 December 2020, becoming the second vaccine approved for use in the national vaccination programme. The first country to issue a temporary or emergency approval for the Oxford-AstraZeneca vaccine was the UK. 

The BBC reported that the first person to receive the vaccine outside of clinical trials was vaccinated on 4 January 2021. As of 29 June 2022, an estimated 24.9 million people have now had at least one dose of this vaccine in the UK, the overwhelming majority without any serious side effects or reactions [2].

A study conducted at Johns Hopkins and published in the New England Journal of Medicine (NEJM) in September 2021 demonstrated that AstraZeneca Plc's (AZN.L) COVID-19 vaccine demonstrated 74% efficacy at preventing symptomatic disease, a figure that increased to 83.5% in people aged 65 and older. The data looked at more than 26,000 volunteers in the United States, Chile, and Peru, who received two doses of the vaccine spaced about a month apart [3].

Looking past the common side effects seen with other vaccinations such as fevers, myalgia, headache, and chills, the most brought-up concern of this vaccine seemed to be vaccine-inducted thrombotic thrombocytopaenia (VITT). A common (one in 10 people) side effect seen in the AZ vaccine was thrombocytopaenia and it is in this pathology, that thrombus formation has been theorised [2].

Up to 29 June 2022, there were 443 reports of people developing rare blood clots which were linked to low platelet levels (thrombocytopaenia) after receiving a dose of the Oxford-AstraZeneca vaccine in the UK. However, here we wanted to highlight the cutaneous effects of the AZ vaccination [2].

A study in September 2021 regarding the clinical and pathological correlation of cutaneous COVID-19 vaccine reactions evaluated for a history of skin biopsy all reports of reactions associated with COVID-19 vaccination identified in an international registry showed that of 803 vaccine reactions reported, 58 (7%) cases had biopsy reports available for review [4]. The most common histopathologic reaction pattern was spongiotic dermatitis, which clinically ranged from robust papules with overlying crust, to pityriasis rosea-like eruptions, to pink papules with fine scales [4].

This patient had developed painful, non-pruritic plaques and papulovesicular lesions, some of which had scales and were de-roofed. Histopathology showed sub-epidermal blistering with negative immunofluorescence ruling out autoimmune bullous disease. Several case reports have suggested the causal link between the Pfizer vaccination and bullous pemphigoid. There have been three known cases and all of them involved a population above 75 years. Other vaccinations such as rabies and swine flu have also been linked to this new-onset bullous pemphigoid phenomenon [5].

## Conclusions

The above case report demonstrates the dermatological effects of the AZ vaccine. It is one of the rarest side effects being reported. As the scientific study is progressing in researching particular side effects due to vaccinations, it is indeed a point to consider the severity shown in this case. Histopathology confirmed the rash to be exfoliative and it was seen in different patterns with the eruption of papules and plaques. The patient was successfully managed after adequate conservative management, but it is worthy to get attention as we continue to vaccinate people because of the effectiveness we have seen in controlling the COVID-19 pandemic. 
